# A Case of Neonatal Lupus Presenting with Myocardial Dysfunction in the Absence of Congenital Heart Block (CHB): Clinical Management and Brief Literature Review of Neonatal Cardiac Lupus

**DOI:** 10.1007/s00246-022-03056-y

**Published:** 2022-12-02

**Authors:** Samhita Jain, Ruggero Spadafora, Sarah Maxwell, Carlos Botas, Hythem Nawaytou, Emily von Scheven, Elizabeth E. Crouch

**Affiliations:** 1 Division of Neonatology, Department of Pediatrics, University of California, San Francisco, San Francisco, CA USA; 2 Department of Pediatrics, University of California, San Francisco, San Francisco, CA USA; 3 Division of Gastroenterology, Hepatology, and Nutrition, Department of Pediatrics, University of California, San Francisco, San Francisco, CA USA; 4 Kaiser Permanente San Francisco Medical Center, Division of Cardiology, Department of Pediatrics, University of California, San Francisco, San Francisco, CA USA; 5 Division of Cardiology, Department of Pediatrics, University of California, San Francisco, San Francisco, CA USA; 6 Division of Rheumatology, Department of Pediatrics, University of California, San Francisco, San Francisco, CA USA; 7 The Eli and Edythe Broad Center of Regeneration Medicine and Stem Cell Research, University of California San Francisco, San Francisco, CA USA

**Keywords:** Neonatal lupus, Left ventricular dysfunction

## Abstract

Neonatal lupus (NLE) is a rare acquired autoimmune disorder caused by transplacental passage of maternal autoantibodies to Sjogren’s Syndrome A or B (SSA-SSB) autoantigens (Vanoni et al. in Clin Rev Allerg Immunol 53:469–476, 2017) which target fetal and neonatal tissues for immune destruction. The cardiac trademark of NLE is autoimmune heart block, which accounts for more than 80% of cases of complete atrioventricular heart block (AVB) in newborns with a structurally normal heart (Martin in Cardiol Young 24: 41–46, 2014). NLE presenting with cardiac alterations not involving rhythm disturbances are described in the literature, but they are rare. Here, we report a case of a neonate with high anti-SSA antibodies who developed severe ventricular dysfunction in the absence of rhythm abnormalities, endocardial fibroelastosis, and dilated cardiomyopathy (Trucco et al. in J Am Coll Cardiol 57:715–723, 10.1016/j.jacc.2010.09.044, 2011), the most common cardiac presentations of NLE. The patient developed severe multiorgan dysfunction syndrome that required prolonged critical care support but fully recovered and was discharged home. We highlight the unusual clinical features of this NLE case and the importance of timely treatment of NLE allowing complete recovery of a critically ill neonate.

**Supplementary Information:**

The online version contains supplementary material available at 10.1007/s00246-022-03056-y.

## Case Report

Our patient was a female neonate born at 35 weeks to a 32-year-old mother who was newly diagnosed with systemic lupus erythematosus (SLE) in the first trimester. The mother tested positive for ANA (titer 1:640), dsDNA (778 IU/ml) and SSA (> 8 AbIndx) antibodies. She received Plaquenil (200 mg twice daily), Atovaquone (1500 mg daily), Azathioprine (150 mg daily), Aspirin (81 mg daily) and Prednisone (35 mg daily) during pregnancy. At the mid-gestation anatomy scan, there were no abnormal findings. Fetus had appropriate growth parameters and had normal heart rate of 140–160 beats per minute with a regular rhythm throughout pregnancy. At 34 gestational weeks, the mother presented with new onset systemic hypertension and premature rupture of membranes. After completing a course of betamethasone, she progressed to preterm labor. The baby was born at 35 weeks via spontaneous vaginal delivery. The Apgar scores were 5, 6 and 8 at 1, 5 and 10 min of life, respectively.

On day of life (DOL) 2, the baby developed new respiratory distress with increased work of breathing and hypoxia, which slightly improved with CPAP. The chest radiograph showed moderate cardiomegaly and pulmonary edema. Laboratory evaluation was remarkable for diffuse end-organ damage with a high blood lactate (5.3 mmol/L), cardiac dysfunction (elevated brain natriuretic peptide > 5000 pg/mL) and myocardial injury (troponin I 0.19 pg/L). She also had significant transaminitis and coagulopathy (Table 1). CBC, electrolytes, and blood gas were within normal limits. Her electrocardiogram and telemetry showed a normal sinus rhythm with no evidence of heart block (CHB) or arrhythmia. The initial echocardiogram showed a structurally normal heart, with severely decreased biventricular function, moderate mitral regurgitation, and no pericardial effusion or coronary abnormalities. There was no echocardiographic evidence of endomyocardial fibroelastosis (EFE). Significant systemic hypertension was recorded since the first day of illness, despite the biventricular dysfunction, requiring multiple continuous infusions of anti-hypertensive medications during the NICU admission.

**Table 1:** Initial laboratory evaluation

	DOL3	DOL 5	DOL9	DOL18	DOL24
BNP (pg/mL)	> 5000	> 5000	> 5000	3460	746
Lactate (mmol/L)	4	4.4	2.8	0.9	0.5
ALT (U/L)	624	660	218	42	
AST (U/L)	2,513	1,919	121		
PT/INR	27.5/2.8	36.5/3.8	15/1.2		
APTT	37.8	79.8	27.5		
Ammonia (umol/L)	98	63	29		
Creatinine (mg/dL)	0.93		0.91	0.45	0.4
Urea (mg/dL)	16		23	41	48
Fibrinogen (mg/dL)				158	230
Ferritin			2940	1908	1440
WBCs/PLT/Hb	8,700/124,000/12.5		37,000/75,000/10.9	19,500/526,000/12.2	

Given the maternal history of SLE and high titers of maternal anti-Ro60 (SSA) antibodies, the index of suspicion for NLE was high. However, due to the absence of NLE specific manifestations like HB, EFE or skin lesions, the initial differential was broad. The perinatal history did not suggest hypoxic fetal insult to the myocardium. Other systemic insults such as sustained hypoglycemia, and hypocalcemia were ruled out by a normal blood gas and basic metabolic panel (Table 1). Thereafter, a comprehensive evaluation for primary myocardial injury including infectious, metabolic and alloimmune etiologies was undertaken. Cultures and titers to investigate infectious agents were all negative. Plasma amino acids, urine organic acids, urine reducing substances and acylcarnitine and carnitine profile values were all within normal limits ruling out the common inborn errors of metabolism. Owing to worsening biventricular cardiac function and hepatic dysfunction during DOL 2–4 (Table 1), the baby was started on NLE treatment without a confirmed diagnosis. The patient received IVIG (1 g/kg) on DOL5 and was started on Methylprednisolone 2 mg/kg BID for 2 weeks, then 1 mg/kg/day BID for an additional 2 weeks [3]. After initiation of therapy for NLE, the cardiac function improved dramatically, with DOL 9 echocardiogram showing normal biventricular function and no mitral regurgitation, with consequently improved systemic perfusion and hepatic function (Table 1). On DOL7, the lupus autoimmune work up resulted with positive SSA antibody and ANA antibody with speckled pattern, confirming the diagnosis of NLE.

Our patient’s clinical course was also complicated by new onset respiratory distress and hypoxemia requiring intubation on DOL 13. The baby received broad spectrum antibiotics (cefepime) and a second dose of IVIG for concerns of Lupus pneumonitis but showed no improvement in her respiratory status. The echocardiogram showed no evidence of cardiac dysfunction or pulmonary hypertension. With appropriate respiratory support and gentle diuresis, the respiratory status improved, and we were able to wean the respiratory support back down to CPAP by DOL 30 and room air by DOL 40. Over the next 2 months, the patient improved slowly. Her anti-hypertensive medications were transitioned to oral clonidine and amlodipine and then weaned off. She was discharged home by 2 months of age with a normal echocardiogram, blood pressure and renal function. She was tapered off hydrocortisone at 4 months of age with a normal subsequent ACTH stimulation test. At the 18-month high-risk infant visit, she displayed a normal neurologic exam and normal development for her age.

## Discussion

CHB is the trademark of NLE and for a long time has been considered the only significant cardiac abnormality in newborns with high titers of anti-RO (SSA) and anti-LA (SSB) antibodies [3]. However, increasing clinical experience has widened the spectrum of cardiac morbidities associated with NLE [7]. Usually, in NLE patients, cardiac abnormalities not involving rhythm disturbances include ventricular dilation and systolic dysfunction, myocardial hypertrophy, and, most commonly, endocardial fibroelastosis (EFE) [3]. The latter manifests on echocardiogram as an echogenicity of the endocardium and has been confirmed in explanted hearts and at autopsy [4]. Overall, these complications are rare, and alone, in absence of rhythm abnormalities, exceptional [5]. Interestingly, one of the few reports that describes cardiac anomalies in absence of CHB is a collection of three cases [4]. Two of the patients died and underwent autopsy, while one received a cardiac transplant. The clinical picture of these patients was dominated by progressive congestive heart failure (CHF) and EFE. Few studies [6, 7] described in their cohorts of CHB patients a subgroup that presented with dilated cardiomyopathy (DCM). In this subgroup there was high mortality even beyond the neonatal period.

Here, we report a unique case of NLE whose clinical course was characterized by a classic picture of heart failure in the absence of complete heart block, EFE or dilated cardiomyopathy. However, contrary to previous reports, our patient showed rapid resolution of the myocardial dysfunction once NLE treatment was initiated. The florid clinical picture in the presence of persistent alloimmune antibody titers suggest a different mechanism causing the transient impairment of the myocardial contractility than the EFE and the DCM syndromes described in literature. We speculate, that in our patient, following the maternal SLE flare in the later stages of the pregnancy, that lupus-related antibodies may have crossed the placenta. However, the degree of cardiac maturation and brevity of the exposure may have prevented the development of CHB and EFE.

To our knowledge this is the first time that isolated and transient cardiac dysfunction in the absence of arrhythmia and EFE is described as the main cardiovascular complication of NLE. This case widens the spectrum of NLE, highlighting the importance of an early and sustained steroidal treatment and demonstrating that, even in patients with multiple organ dysfunction, a sustained positive long-term result can be achieved with timely intervention.

## Supplementary Information

Below is the link to the electronic supplementary material.

Supplementary file1 (MP4 349 KB)

Supplementary file2 (MP4 326 KB)
